# Variabilities of hydatidosis in domestic animals slaughtered at Cairo and Giza abattoirs, Egypt

**DOI:** 10.14202/vetworld.2019.998-1007

**Published:** 2019-07-11

**Authors:** Faten A. M. Abo-Aziza, Samah S. Oda, Dina Aboelsoued, T. K. Farag, Abdulaziz M. Almuzaini

**Affiliations:** 1 Department of Parasitology and Animal Diseases, Veterinary Research Division, National Research Centre, Cairo, Egypt; 2 Department of Pathology, Faculty of Veterinary Medicine, Alexandria University, Edfina, Egypt; 3 Department of Veterinary Medicine, College of Agriculture and Veterinary Medicine, Qassim University, Buraydah, Saudi Arabia

**Keywords:** abattoirs, antioxidant activity, hydatidosis, prevalence

## Abstract

**Aim:**

The effect of some variables on hydatidosis in animals slaughtered at Cairo and Giza abattoirs was investigated and the influence on serum biochemical parameters, antioxidant enzymes, and histopathological lesions caused by these parasites as a consequence was estimated.

**Materials and Methods:**

The effect of some variables on hydatidosis in 397 sheep, 401 cattle, 435 buffaloes, and 341 camels slaughtered at Cairo and Giza abattoirs was investigated, and the influence on serum biochemical parameters, antioxidant activity and histopathological lesions caused by these parasites as a consequence was estimated.

**Results:**

The results revealed that 39 sheep (9.8%), 74 cattle (18.4%), 95 buffaloes (21.8%), and 79 camels (23.25%) were infected. Concerning age variations, 165 young and 232 adult sheep, 215 young and 186 adult cattle, 194 young and 241 adult buffaloes, and 112 young and 229 adult camels were examined. The prevalence of hydatidosis was higher in adult sheep, cattle, and camel; 32 (13.8%), 49 (26.3%), and 56 (24.5%) than the younger ones 7 (4.2%), 25 (11.6%), and 23 (20.5%), respectively. Two hundred and eighty-eight sheep, 171 cattle were examined during winter. However, 109 sheep, 230 cattle were examined during summer. Hydatidosis infection in sheep and cattle was higher in winter 26 (9.01%) and 47 (27.5%) than in summer 13 (11.9%) and 27 (11.7%), respectively. Out of 133 sheep and 128 camels slaughtered in El-Basatin abattoirs, 36 (15.3) and 38 (29.7%) showed higher prevalence than that from El-Warak and El-Moneib abattoirs. Comparing with the non-infected groups, alkaline phosphatase activity decreased in hydatid-infected animals, while cholesterol and liver enzymes activities increased. Total lipid and triglyceride levels decreased in infected camels. Glutathione peroxidase, superoxide dismutase, and catalase decreased in hydatid-infected animals.

**Conclusion:**

The disturbance in the biochemical parameters, liver enzymes, and the antioxidant activities was consistent with the pathological findings that indicated the risk of hydatidosis infection. Finally, this study clarified the variabilities of hydatidosis in Cairo and Giza abattoirs as a starting point for future studies in different regions in Egypt.

## Introduction

Hydatidosis is an important disease caused by *Echinococcus* species animal parasites [1]. The larval stage of the small taeniid-type tapeworm (*Echinococcus granulosus*) may cause infection in herbivorous animals and humans causing hydatid disease cystic echinococcosis (CE) [2]. Hydatid cysts can be spread in different organs of host such as liver, lung, heart, and brain that may result in death [3]. Hepatic hydatidosis injuries in livestock animals cause economic loss due to the condemnation of tissues. *E. granulosus* eggs spread by infected dog feces into the environment and eggs are able to stay alive in soil, water, and vegetables and can survive in snow and freezing conditions. The eggs are highly resistant to environmental stress and can live at least for a year in the environment [4]. Stray dogs may provide favorable conditions to circulate this parasite and the spread of epidemic foci [5]. Stray dogs are usually roaming near abattoirs and on streets, feeding on carcasses of the dead animals in rural areas. Stray dogs can also have free access to fields of domestic animals and yards, contaminating the environment with the eggs of *Echinococcus* [6].

Many variables can affect hydatidosis such as regions, sex, age, and seasons. Temperate zones including several parts of the Mediterranean regions, central and Southern parts of Russia, some parts of America, Australia, North and East Africa, China, and central Asia recorded the highest prevalence of hydatidosis in human and animal hosts [7]. Hydatidosis is currently considered an endemic zoonotic disease in the Mediterranean region [8]. Hydatidosis is endemic in all North African countries including Algeria, Morocco, Tunisia, Libya, and Egypt [9] and also in sub-Saharan Africa including Mauritania, Tanzania, Sudan, Kenya, and Ethiopia [10].

Hydatidosis may vary from country to country or even within a country [11]. Furthermore, hydatidosis is associated with variable species of animals [12]. In Morocco, hydatidosis prevalence was 10.58% in sheep, 12.03% in camels, and 22.98% in cattle [13]. In Algeria, the infection rate was 13.9% in cattle and 24.8% in camels [14]. In Tunisia, the incidence of hydatidosis reached 10.1% of camels [15] and 40% of sheep [16]. In Libya, infection rates in livestock ranged from 1.0% to 13.9% in cattle, 1.7% to 33.4% in sheep, and 1.4% to 40.0% in camels [17]. In Iran, the hydatid cysts in cattle and sheep recorded 15-20% and 20-30%, respectively [18]. In Ethiopia, hydatidosis rate was 7.03% and 42.86% in sheep and cattle, respectively [12]. Egypt started to be considered as an emerging endemic area for hydatidosis [19], and the World Health Organization has included hydatidosis as part of a Neglected Zoonosis subgroup in its control of neglected tropical diseases 2008-2015 strategic plans [20]. There are deficient studies concerning the effect of different variabilities on hydatidosis in Egypt.

The present study was investigated in Cairo and Giza in Egypt to be a starting point. The effect of some variables on hydatidosis in sheep, cattle, buffaloes, and camels slaughtered at Cairo and Giza abattoirs was investigated, and the influence on serum biochemical parameters and histopathological lesions caused by these parasites as a consequence was estimated. As well as, glutathione peroxidase (GSH-PX), superoxide dismutase (SOD), and catalase (CAT) activities in serum samples were measured as a means of assessing the oxidative stress during infection.

## Materials and Methods

### Ethical approval

This study was approved by Institutional Animal Care and Use Committee of National Research Centre (Protocol Number: 16/230), Cairo, Egypt.

### Study design, animals, and antemortem inspection

A study of cross-sectional type was conducted from December 2016 to October 2017 to determine the prevalence besides the biochemical and histopathological alterations of hydatidosis-induced lesions in liver and lung of farm animals slaughtered at Cairo and Giza abattoirs (El-Warak, El-Basatin, and El-Moneib abattoirs). The samples were collected from 397 sheep, 401 cattle, 435 buffaloes, and 341 camels after slaughtering according to the governmental regulations. The slaughtered animals were of different ages, sexes and originated from different areas of the country. List of the animals to be slaughtered was prepared and pre-slaughter examinations of sheep, cattle, buffaloes, and camels were conducted to determine the age and sex of animals. Animals were classified according to the dentition. Animals, which were without permanent incisor teeth, were considered as young with average age about 1-1.5 years, while those having one or more permanent pair of incisor teeth were considered adults with average age over 2 years.

### Sampling

Using systematic random sampling methods, blood samples of slaughtered animals were collected immediately after slaughter. The blood was collected in plain tubes and allowed to clot for serum extraction by centrifugation of clotted blood at 3000 rpm/15 min then divided in small aliquots and stored at −20°C for biochemical analysis. The corresponding livers and lungs of the slaughtered animals were examined by naked eyes and tissue specimens were collected from all slaughtered animals for microscopical and histopathological examination.

### Postmortem examination

Livers and lungs were thoroughly inspected by applying the routine meat inspection procedures during postmortem examination [21]. Palpation and many incisions were made through each liver and lung. Then, careful visual examination and identification for the presence of cysts and other abnormalities were carried out [22], and the results were recorded. Five minutes was spent in postmortem examination for each animal.

### Histopathological examination

The collected tissue specimens from affected livers were quickly fixed for at least 24 h in 10% neutral buffered formalin. Fixed specimens were trimmed, washed, and dehydrated in ascending grades of ethyl alcohol, then cleared in xylene and embedded in paraffin wax. Thin sections of 4-5 µm thickness were performed and stained with hematoxylin and eosin for general microscopic examination according to the method described by Bancroft *et al*. [23]

### Biochemical studies

Serum total protein (TP) was determined by the colorimetric method (Biuret reagent) using SPECTRUM kits (BioMerieux, SA). Albumin (ALB) was determined by a modified bromocresol green colorimetric method using SPECTRUM kits (BioMerieux, SA). Globulin (GLB) and ALB/GLB (A/G) ratio were calculated. Determination of lipogram biochemical values was performed by enzymatic colorimetric method using Linear Chemicals kits to measure serum total lipid, triglycerides (TG), and cholesterol (CHOL). Various liver enzymes such as alanine aminotransferase (ALT), aspartate aminotransferase (AST), and alkaline phosphatase (ALP) were measured using ultraviolet enzymatic colorimetric method as the standardized method using Linear Chemicals S.L. kits. Colorimetric reaction was measured using spectrophotometer.

### Antioxidant activity

GSH-PX, SOD, and CAT activities in serum samples were assessed using SPECTRUM kits (BioMerieux, SA). Absorbances were measured at 340 nm, 560 nm, and 520 nm, respectively, by spectrophotometer.

### Statistical analysis

Descriptive statistics such as percentage were calculated and the prevalence of hydatidosis in sheep, cattle, buffaloes, and camels of different age, sex, season, and origin. Groups were evaluated by Pearson’s Chi-square (*χ*^2^) test and differences were considered statistically significant if p<0.05. The data of biochemical study and the antioxidant activities were statistically analyzed by Student’s t-test at level p<0.05 [24] using Statistical Package for the Social Science (IBM, USA) for Windows version 15 computer program.

## Results

### Postmortem examination

**Figure-1:**
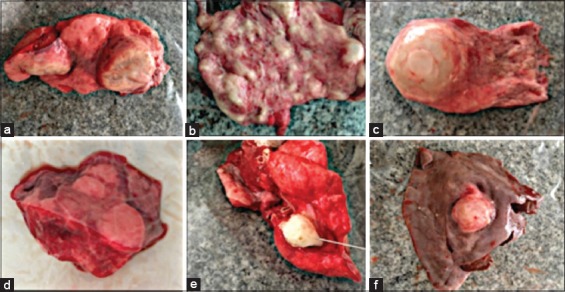
Gross pathological changes caused by hydatidosis; (a) multiple hydatid cysts. (b) Abscess all over the lung parenchyma. (c) Tumor-like fluid-filled cysts, surrounded by a fibrous wall. Hydatid lung cysts may be varied in diameter between 1 and 20 cm. Large cysts can shift the mediastinum causing atelectasis of adjacent parenchyma. (d) Palpated hydatid cyst in lung of infected cattle showing red hepatization indicating pneumonia. (e) Semi-calcified cysts with a hard wall surrounding cheese-like material in the lung of infected sheep. (f) Liver of sheep with hydatid cyst end to be located in the right lobe. Cyst size in the liver is variable between 1 mm and 5 mm in diameter.

Figure-1 showed gross pathological changes caused by hydatidosis. In the lung of infected camel, there was multiple hydatid cysts (Figure-1a), abscess in all over the lung parenchyma (Figure-1b), and tumor-like fluid-filled cysts and surrounded by a fibrous wall (Figure-1c). The size of these lung cysts was variable, ranging from 1 to 20 cm in diameter. In the lung of hydatidosis-infected cattle, palpated hydatid cysts were found with red hepatization indicating pneumonia (Figure-1d). Semi-calcified cysts with a hard wall surrounding cheese-like material were also found in the lung of sheep (Figure-1e). Liver of infected sheep showed hydatid cyst end to be located in the right lobe (Figure-1f). Moreover, cyst size in the liver of cattle and camels was variable from 1 mm to 5 mm in diameter.

### Variables of hydatidosis prevalence

**Figure-2:**
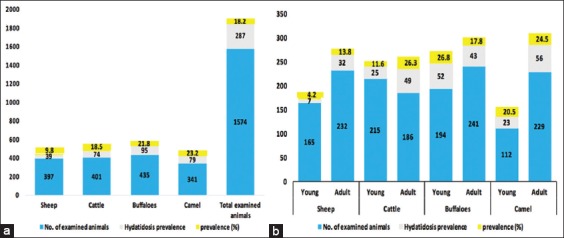
Univariable species affecting hydatidosis (a) Univariable age affecting hydatidosis (b) χ^2^ test; (p<0.05).

Macroscopic and microscopic examination of cysts isolated from the livers and lungs revealed that of 1574 examined animals included 397 sheep, 401 cattle, 435 buffaloes, and 341 camels, there were 287 (18.2%) hydatidosis infected. In particular, 39 sheep (9.8%), 74 cattle (18.5%), 95 buffaloes (21.8%), and 79 camels (23.2%) were hydatidosis infected (Figure-2a).

Concerning age variations, 165 young and 232 adult sheep, 215 young and 186 adult cattle, 194 young and 241 adult buffaloes, and 112 young and 229 adult camels were examined. Higher hydatidosis prevalence was recorded in adult sheep, cattle, and camel 32 (13.8%), 49 (26.3%), and 56 (24.5%) than the younger ones 7 (4.2%), 25 (11.6%), and 23 (20.5%), respectively. However, no significant difference was observed in the hydatidosis prevalence between both ages in buffaloes (Figure-2b).

**Figure-3:**
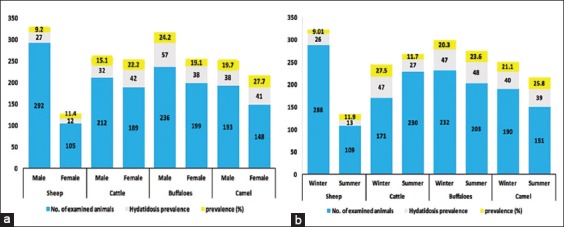
Univariable sex affecting hydatidosis (a) Univariable season affecting hydatidosis (b) Winter: From December 2016 to April 2017. Summer: From May to October 2017. χ^2^ test; (p<0.05).

As shown in Figure-3a, there were no significant differences in the prevalence rates of hydatidosis in both sex groups among sheep, cattle, buffaloes, and camels.

Regarding seasonal prevalence, 288 sheep, 171 cattle, 232 buffaloes, and 190 camels were examined during winter. However, 109 sheep, 230 cattle, 203 buffaloes, and 151 camels were examined during summer. The results revealed that hydatidosis infection in sheep and cattle was higher in winter 26 (9.01%) and 47 (27.5%) than in summer 13 (11.9%) and 27 (11.7%). However, there was no significant difference in the prevalence rate of hydatidosis in buffaloes and camel in both seasons (Figure-3b).

**Figure-4:**
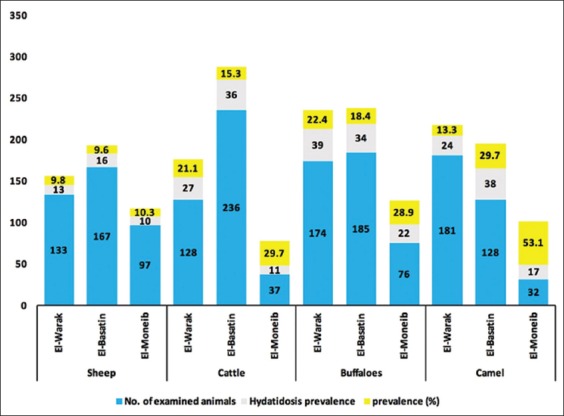
Univariable abattoirs affecting hydatidosis. χ^2^ test; (p<0.05)

There were 133 sheep, 128 cattle, 174 buffaloes, and 181 camels slaughtered in El-Warak abattoir. However, 167 sheep, 236 cattle, 185 buffaloes, and 128 camels were slaughtered in El-Basatin abattoir. Moreover, 97 sheep, 37 cattle, 76 buffaloes, and 32 camels were slaughtered in El-Moneib abattoir. It was recorded that the prevalence of hydatidosis was higher in sheep from El-Basatin; 16 (9.6%) than that from El-Warak; 13 (9.8%) and El-Moneib; 10 (10.3%) abattoirs. Likewise, hydatidosis prevalence in cattle from El-Basatin; 36 (15.3) was higher than that from El-Warak; 27 (21.1) and El-Moneib; 11 (29.7) abattoirs. However, the prevalence of hydatidosis was higher in buffaloes from El-Warak abattoir; 39 (22.4%) while it was higher in camel from El-Basatin abattoir; 38 (29.7%) than their corresponding from other abattoirs (Figure-4).

### Histopathological studies

**Figure-5:**
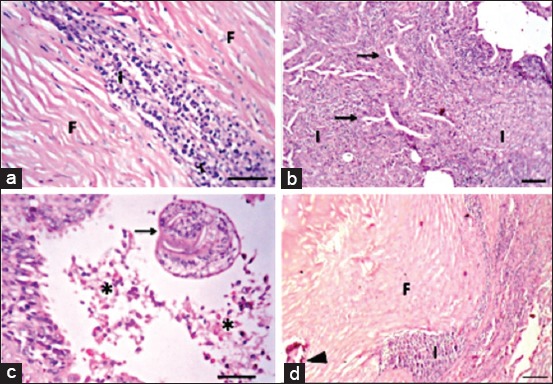
Lung of hydatidosis-infected camel showing: (a) Hydatid cyst wall in lung of camel showing fibrous layer (F) and inflammatory reaction (I), Bar=50 µm. (b) Thickening of interstitium with inflammatory cells with fertilization of alveolar epithelium (arrows), Bar=100 µm. (c) Presence of scolex (arrow) with necrotic debris (asterisk) inside the bronchiolar lumen, (Bar=50 µm). (d) Lung of sheep showing hydatid cyst wall. Fibrous layer (F), inflammatory reaction (I), and calcification (arrowhead). Hematoxylin and eosin (HE), Bar=100 µm (All HE).

**Figure-6:**
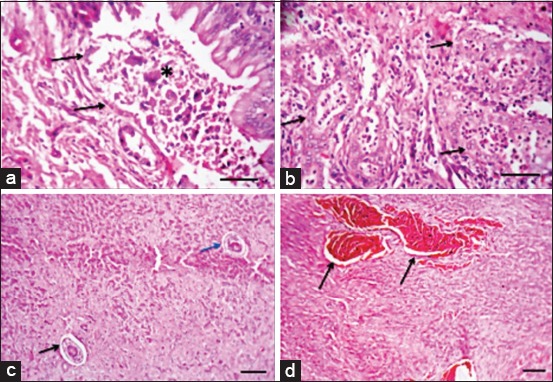
Liver of hydatidosis-infected camel showing: (a) Necrosis of biliary epithelium (arrows) with the presence of necrotic debris inside the lumen (asterisk), Bar=50 µm. (b) Presence of numerous neutrophils inside the lumen of bile ductless (arrows), Bar=50 µm. Liver of hydatidosis-infected cattle showing: (c) Massive cirrhosis with atrophied hepatic parenchyma and presence of scolices in portal vein (black arrow) and central vein (blue arrow), Bar=100 µm. (d) Severe congestion (arrows), Bar=100 µm (All hematoxylin and eosin).

Lungs of camels had characteristic hydatid cysts wall that was consisted of outer laminated layer and fibrous layer infiltrated with inflammatory cells such as lymphocytes, macrophages, and plasma cells (Figure-5a). Some lung sections showed cystic alveolar dilatation that was impacted with inflammatory cells, particularly lymphocytes, macrophages, plasma cells, and few neutrophils. There was severe thickening of interstitium with mononuclear inflammatory cells and granulomas formation with fertilization of the alveolar epithelial cells (Figure-5b). Scolices were found in alveoli and bronchioles with mononuclear inflammatory cells and few multinucleated giant cells aggregation (Figure-5c). Similar findings were detected in lungs of sheep, besides multifocal areas of calcification along the cyst wall (Figure-5d). In sheep liver, biliary epithelial necrosis (Figure-6a) and numerous neutrophils in bile ductless were observed (Figure-6b) with marked portal inflammatory reaction. Scolices were present in portal and central veins with massive cirrhosis (Figure-6c) and severe congestion was observed in cattle liver (Figure-6d).

### Biochemical studies

Protein profile in serum of hydatidosis infected and non-infected sheep, cattle, buffaloes, and camels was analyzed. It was found that TP and ALB decreased in hydatid infected sheep, cattle, buffaloes, and camels, while GLB and A/G ratio increased compared with their corresponding non-infected groups (Table-1). Serum lipid profile and liver enzymes (ALT and AST) of hydatid infected and non-infected sheep, cattle, buffaloes, and camels were determined (Table-1). In hydatid-infected sheep, CHOL level was showed significant elevation (p<0.01) than non-infected group. As well as, the results of liver enzymes activities in hydatid-infected sheep, cattle, buffaloes, and camels showed significant elevation (p<0.05) comparing with their corresponding non-infected groups. Results of lipid profile showed that the level of total lipid decreased while CHOL level increased (p<0.01) in hydatid-infected buffaloes comparing with the non-infected group. Moreover, the level of total lipid and TG of hydatid-infected camels decreased (p<0.05) while CHOL level increased (p<0.01) comparing with the non-infected group. However, a decline in ALP activity than non-infected group was recorded in all animal species except sheep (Table-1).

**Table 1:** Protein and lipid profiles and liver enzymes in serum of hydatidosis infected and non-infected sheep, cattle, buffaloes, and camels.

Parameter	Animals							

	Sheep		Cattle		Buffaloes		Camels	
			
	Non-infected	Infected	Non-infected	Infected	Non-infected	Infected	Non-infected	Infected
TP(gm%)	7.35±0.44	5.64±0.24*	6.48±±0.375	5.34±0.49^NS^	7.93±0.439	5.78±0.390*	6.75±0.72	6.36±0.18^NS^
ALB(gm%)	4.83±0.39	2.64±0.396*	4.71±0.374	3.19±0.198*	4.39±0.165	3.01±0.385*	4.59±0.37	2.61±0.45*
GLB(gm%)	3.75±0.026	4.34±0.14*	3.28±0.203	4.58±0.42*	3.51±0.25	4.89±0.386**	3.02±0.26	4.97±0.36**
A/G ratio	1.21±0.08	1.51±0.02*	1.05±0.091	1.29±0.05^NS^	1.13±0.135	1.96±0.015**	1.11±0.03	1.66±0.11**
Total lipid	56.39±3.57	48.62±2.8^NS^	62.27±3.46	49.85±4.15^NS^	57.61±4.09	36.23±3.73**	54.84±4.54	36.78±3.16*
TG(mg%)	46.36±2.56	43.86±3.46^NS^	44.61±2.68	39.31±2.24^NS^	42.82±3.84	36.72±2.87^NS^	43.73±3.09	31.25±2.75*
CHOL(mg%)	52.77±5.24	87.46±4.51**	49.58±3.42	54.73±2.33^NS^	79.58±6.42	123.32±6.54**	65.43±3.62	112.53±4.91**
ALT(U/L)	27.83±3.34	41.37±2.86*	26.74±3.26	43.05±2.17**	28.13±2.87	37.45±2.16*	27.35±2.74	40.95±3.46*
AST(U/L)	116.25±7.85	154.11±8.13*	121.34±5.63	138.92±3.54*	135.34±5.63	176.35±8.42**	125.34±5.33	218.84±12.87**
ALP(U%)	32.64±3.79	25.68±3.28^NS^	47.52±3.49	29.43±2.84**	42.09±3.51	26.48±2.72*	34.57±3.29	23.95±2.37*

In the same species;

*,** mean significant difference than non-infected group at P<0.05 and P<0.01, respectively. NS=Non-significant. ALP=Alkaline phosphatase, TP=Total protein, ALB=Albumin, GLB=Globulin, CHOL=Cholesterol, ALT=Alanine aminotransferase, AST=Aspartate aminotransferase

### Antioxidant activity

The effect of hydatidosis on serum GSH-PX, SOD, and CAT activities in sheep, cattle, buffaloes, and camels was studied. It was found that GSH-PX (p<0.05) and SOD (p<0.01) decreased in hydatid-infected sheep than non-infected group. However, CAT activity decreased (p<0.05) in hydatid-infected cattle, while GSH-PX decreased (p<0.01) in hydatid-infected buffaloes comparing to non-infected groups. Moreover, GSH-PX (p<0.05), SOD (p<0.01), and CAT (p<0.05) serum activities were lower in hydatid-infected camel than non-infected group (Table-2).

**Table 2:** Serum GSH-PX, SOD, and CAT activities in hydatidosis infected and non-infected sheep, cattle, buffaloes, and camels.

Parameter	Animals							

	Sheep		Cattle		Buffaloes		Camel	
			
	Non-infected	Infected	Non-infected	Infected	Non-infected	Infected	Non-infected	Infected
GSH-PX(U/ml)	8.78±1.64	4.37±0.58*	7.45±1.38	4.23±0.71^NS^	8.61±0.47	3.59±0.91**	7.54±0.92	3.14±0.86*
SOD(U/ml)	109.67±7.81	73.48±5.36**	110.32±11.07	82.51±4.92^NS^	112.83±9.22	93.76±5.34^NS^	124.36±9.74	78.25±4.13**
CAT(U/ml)	158.61±9.28	134.32±9.52^NS^	137.38±9.12	103.62±8.33*	141.38±5.44	128.41±7.18^NS^	158.61±8.41	118.93±7.66*

In the same species;

*,** mean significant difference than non-infected group at P<0.05 and P<0.01, respectively. NS=Non-significant. GSH-PX=Glutathione peroxidase, SOD=Superoxide dismutase, CAT=Catalase

## Discussion

In the present study, variable factors of hydatidosis infection in sheep, cattle, buffaloes, and camels slaughtered at Cairo and Giza abattoirs were investigated, and the influence on serum biochemical parameters, antioxidant activities, and histopathological lesions produced as consequently by this parasitic infection was assessed. Hydatidosis prevalence was varied according to species. It was significantly higher in camel (23.2%) followed by buffaloes (21.8%), then cattle (18.5%) and sheep (9.8%). These results agreed with Ibrahim [25], who found that camels were reported as the domestic intermediate host most likely to be infected; however, the highest cyst intensity was recorded in cattle. Our results disagreed with Fromsa and Jobre [26] who found that cattle were identified to have the highest prevalence of CE. Nonga and Karimuribo [27] supported another opinion that small ruminants had frequently been recorded high infection rates. Among Egyptian ruminants, hydatidosis prevalence was 2.53% and 0.3% in camels and sheep, respectively, and was 6.4% cattle in Mansoura official abattoirs [28]. Concerning animals from different abattoirs of Upper Egypt, it was found that sheep were the most affected by hydatidosis (14.1%), while the prevalence in camels was 5% and was 0.068% in cattle [29]. These variations in the prevalence could be due to many factors such as the origin of the slaughtered animals, the strain difference of *E. granulosus*, the difference in sample size, the difference in animals’ geographical distribution, and the selection of the studied animals and also, variability could be related to age factors [29]. Other factors such as dogs might be responsible for high prevalence of hydatidosis [6].

Concerning age variability, it was observed that the prevalence of hydatidosis was higher in adult sheep, cattle, and camel. This is in agreement with the previous study findings [30]. Otero-Abad and Torgerson [31] stated that the age of the host became an infection determinant for many farm host species. Small ruminants aging 3 years or older have risk of 1.6 times more than younger animals [32]. An increase of cyst abundance was observed in farm animals with the advancement of age [25]. This difference in the prevalence between older and younger animals could be mainly due to longer exposure time to *Echinococcus* eggs [33]. However, Shimeles and Awole [12] found that no significant differences were recorded between young and adult ruminants in the number of cysts and prevalence based on age.

The prevalence of cystic parasitic infection in all examined animals was higher in winter than summer. This result parallels to the previous study by Ardo *et al*. [34]. Hydatidosis prevalence due to seasonal variations was recorded through abattoir meat inspection [25]. Fromsa and Jobre [26] found that the number of births in each season, as well as the environmental factors such as high altitudes and increasing annual rainfall, was found to affect the rate of hydatidosis in livestock.

Statistical analysis of the prevalence of hydatidosis according to sex variations revealed that sex had no influence on the rate of infection that both sex groups among sheep, cattle, buffaloes, and camels were equally susceptible to hydatidosis. The previous studies that agreed with the present results were reported by Bayu *et al*. [30]. Others supported the opinion that females had a higher rate of hydatidosis infection than males [25,35]. Females were also reported to have higher prevalence than males in Iran [36], Eastern Libya [17], and Northwestern Ethiopia [37]. Otero-Abad and Torgerson [31] identified the gender of the host as a possible determinant of hydatidosis prevalence. The explanation behind that might be because female animals were not slaughtered in younger ages and they were sent to abattoirs after getting calves and milking for years and so, another factor which is animal aging could also affect the infection rate in this situation [33]. However, Castiglia *et al*. [38] and Erbeto *et al*. [39] reported a higher prevalence of hydatidosis in males than females in Italy and Ethiopia, respectively.

The prevalence of parasitic infection was higher in sheep, cattle, and camels coming from El-Basatin abattoir followed by El-Warak abattoir than that coming from El-Moneib abattoir. The high prevalence obtained in this work might be probably due to conducive environment, presence of intermediate host, and high number of disease reservoirs (cattle, goat, and sheep) [40]. High prevalence of parasitic infection in animal’s samples coming from El-Basatin abattoir could be due to high population in this abattoir which might lead to imperfection in control programs systems [41] and lack of suitable elimination of infectious carcasses [42]. As well as, the slaughtered animals might be come from various areas of different climatologic conditions, in addition to, deficiency of farmers’ awareness [41].

Histopathologic examination in the present investigation revealed that scolices of the parasite were seen in lung and liver sections of hydatid-infected animals. Cellular infiltrations were mostly consisted of mononuclear cells such as lymphocytes, macrophages, and plasma cells and neutrophils. Eosinophils were not prominent in the lung and liver sections. The cellular infiltrates were multifocal, diffuse, or in the form of granulomas. The cellular reaction associated with hydatid cysts was indicative of delayed hypersensitivity reaction [43]. The presence of numerous neutrophils in alveoli and bile ductless indicated secondary bacterial infection. Degeneration and necrosis of hydatid cysts appeared as suppuration, caseation, and calcification. Lesions associated with hydatid cysts were similar to those reported previously [44].

The functional status of liver was explored by the determination of serum biochemical analysis. The results of this work indicated that the mean values of serum TP and ALB were significantly lower in hydatid-infected sheep, cattle, buffaloes, and camels. However, the GLB levels and A/G ratio of these infected animals were significantly higher compared with non-infected groups. These results could be referred to as hepatic injuries and degeneration associated with hypoxia [45]. As well as, the decline in serum TP and ALB levels besides the elevation in GLB levels might be a result of parasitic infection-induced stresses [46]. In addition, this hyperglobulinemia in hydatid-infected animals could be considered as a response to the parasitic antigen which might be a result of immune system stimulation and production of immunoglobulins against infection [47].

Results of lipid profile of this investigation had that hydatid-infected sheep had significantly higher CHOL level. However, hydatid-infected cattle did not show any significant difference in lipid profile than the non-infected ones. As well as, the hydatid-infected buffaloes showed significantly lower level of total lipid besides higher CHOL level than their corresponding non-infected group. Similarly, the results of lipid profile in hydatid-infected camels showed significantly lower level of total lipid and TG and significantly higher CHOL level comparing with the non-infected camels. Hypercholesterolemia was detected previously in several parasitic infected animals [48]. It was reported that parasite infection in camel could lead to lipid metabolism deviations [49].

The hepatic enzymes were considered the main indicator that primarily characterizes hepatocellular necrosis and cholestasis; therefore, they had a distinct efficacy in the diagnosis of severe liver disorders [44]. In this work, the results of liver enzymes activities in hydatidosis-infected sheep, cattle, buffaloes, and camels showed a significant elevation in the ALT and AST activities comparing with their corresponding non-infected groups. However, activity of ALP was significantly decreased in hydatid-infected cattle, buffaloes, and camels comparing to their corresponding non-infected groups. It was clear that estimation of serum activities of these specific enzymes in the infected animals could help in the diagnosis of hydatid cyst [44]. Little information known about serum biochemical profiles of liver hydatidosis had to consider that these results might be due to the elevation in *in vivo* liver enzymes activity due to chronic granulomatous hepatitis in ruminants with hydatid cyst or might be due to metabolic disturbances. The higher activities of ALT and AST found in our investigation might be a result of hepatocellular damage as previously recorded by Hodžić *et al*. [50]. Gonzalo-Orden *et al*. [51] reported that activity of AST returned to the normal values 11-week post-infection. Another study reported that AST activity was significantly lower in serum of infected sheep [52].

Estimation of serum enzymatic antioxidant activities in parasitic infection such as GSH-PX, SOD, and CAT were means of assessing the oxidative stress [53]. In this work, GSH-PX and SOD serum activities decreased significantly in hydatidosis infected sheep, while CAT activity significantly decreased in hydatidosis-infected cattle and buffaloes. However, GSH-PX, SOD, and CAT serum activities were lower in hydatidosis-infected camels. The data reflected the relationship between hydatidosis infection in farm animals and the oxidative stress [54].

## Conclusion

Variable factors are affecting hydatidosis prevalence such as animal species, age, seasonal variations, and housing as well as management systems in different areas under investigation. The disturbances in the biochemical blood parameters, liver functions, and the antioxidant activities with the pathological changes could be used as complementary in the diagnosis of hydatidosis. Finally, this study clarified the variabilities of hydatidosis in Cairo and Giza as a starting point to studies in different regions in Egypt in the future.

## Authors’ Contributions

FAMA designed the study, performed the biochemical and antioxidant activity analysis and contributed to laboratory work analysis and data interpretation, manuscript preparation, and corresponded the authorship. SSO performed the pathological examination. DA performed the macroscopical examination, shared in data interpretation, and further assisted in the manuscript preparation. TKF contributed to sample collection and shared in laboratory work analysis. AMA shared in laboratory work analysis, data interpretation, and manuscript preparation. All authors have read and approved the final manuscript.
